# Is flexible sigmoidoscopy screening associated with reducing colorectal cancer incidence and mortality? a meta-analysis and systematic review

**DOI:** 10.3389/fonc.2023.1288086

**Published:** 2023-12-13

**Authors:** Xinmiao Wang, Luchang Cao, Xiaotong Song, Guanghui Zhu, Baoyi Ni, Xinyi Ma, Jie Li

**Affiliations:** ^1^ Department of Oncology, Guang’anmen Hospital, China Academy of Chinese Medical Sciences, Beijing, China; ^2^ Graduate School, Beijing University of Chinese Medicine, Beijing, China

**Keywords:** colorectal cancer, flexible sigmoidoscopy, incidence, mortality, screening

## Abstract

**Background:**

The question of whether flexible sigmoidoscopy (FS) for colorectal cancer (CRC) affects incidence or mortality remains unclear. In this study, we conducted a meta-analysis and systematic review to explore this issue.

**Methods:**

A systematic search of *PubMed*, *EMBASE*, and *ClinicalTrials.gov* was performed for cohort studies (CS), case–control studies, and randomized controlled trials (RCTs) of people who underwent FS and reported mortality or incidence of CRC until 11 December 2022. Relative risk (RR) was applied as an estimate of the effect of interest. To combine the RRs and 95% confidence intervals, a random-effects model was used. The quality of the included studies and evidence was assessed by the Newcastle-Ottawa quality assessment scale, the Jadad scale, and the “Grading of Recommendations Assessment, Development and Evaluation System.”

**Results:**

There were a total of six RCTs and one CS, comprising 702,275 individuals. FS was found to be associated with a 26% RR reduction in CRC incidence (RR, 0.74; 95% CI, 0.66–0.84) and a 30% RR reduction in CRC mortality (RR, 0.70; 95% CI, 0.58–0.85). In the incidence subgroup analysis, FS significantly reduced the incidence of CRC compared with non-screening, usual care, and fecal immunochemical testing. Significance was also shown in men, women, distal site, stages III–IV, ages 55–59, and age over 60. In terms of the mortality subgroup analysis, the results were roughly the same as those of incidence.

**Conclusion:**

According to this study, FS might reduce the incidence and mortality of CRC. To confirm this finding, further prospective clinical studies should be conducted based on a larger-scale population.

**Systematic review registration:**

https://www.crd.york.ac.uk/prospero/, identifier CRD42023388925.

## 1 Introduction

In the digestive system, colorectal cancer (CRC) is the most common malignant tumor, ranked third in incidence and second in mortality according to the “Global Cancer Statistics 2020,” which poses a serious threat to human health (1). According to the American Cancer Society, CRC had the third highest death rate among men and women in 2022 (2). Over time, the environment and lifestyle of people have changed quitely. In addition to smoking (3), consumption of red and processed meat (4), obesity, and lack of exercise (5) are also associated with CRC. An even more ominous statistic is that 2.5 million cases of CRC will occur in the world by 2035 (1, 6). The early symptoms of CRC are not obvious and specific, and most patients are usually diagnosed in the advanced stages (7). In the vast majority of cases of CRC, the process of “normal mucosa–adenomatous polyp–polyp canceration–invasion and metastasis” takes place over the course of 10 years (8). As a result, the primary and secondary prevention of CRC must be prioritized.

Lower gastrointestinal endoscopy, especially colonoscopy and sigmoidoscopy, has been reported to detect and clamp precancerous lesions, which plays an important role in early detection (9, 10). In many countries, precancerous screening has become more popular (11, 12). According to the United States Preventive Medicine Task Force (USPSTF), CRC screening should be offered to people aged 50 to 75 years (13). The most common screening methods for CRC are occult blood (14), exfoliated DNA tests (15), flexible sigmoidoscopy (FS) (16), colonoscopy (17), and computed tomographic colonography (18). Endoscopic examination can provide a visual view of the intestinal mucosa; however, early preparation for a colonoscopy can be cumbersome and costly, and there is also the risk of complications associated with the procedure itself. In contrast to colonoscopy, the FS examination has a much higher sensitivity (19) and lower examination risk (3.4/10,000 vs. 2.8/1,000) (20–22). In addition, most lesions within the distal colon covered by FS can be removed during screening, making FS screening both diagnostic and therapeutic. Therefore, FS has a significant clinical importance for the diagnosis and screening of CRC. Systematic reviews have been conducted to evaluate the contribution of FS to the incidence and mortality of CRC in 2013 and 2014 (23, 24). Several new studies have recently been published that examine the effect of FS on CRC, but no definitive conclusion has been reached (25–31). The aims of this study are to include high-quality randomized controlled trials, focusing on the effects of factors such as sex, age, tumor location, screening methods, follow-up methods, and geography; objectively evaluate the role of FS in the incidence and mortality of CRC, as well as evaluate the quality of synthesized evidence, based on current evidence; and hopefully provide evidence-based data for the clinical indications of FS screening in the CRC guidelines.

## 2 Methods

### 2.1 Search strategy

This study was registered in PROSPERO with the number “CRD42023388925” and reported based on the “Meta-analysis of Observational Studies in Epidemiology” (MOOSE) and the “Preferred Reporting Items for Systematic Reviews and Meta-Analysis” (PRISMA) (32). A team of two reviewers (LC and XS) independently searched in *PubMed*, *EMBASE*, and *ClinicalTrials.gov* since the inception of the database until 11 December 2022. The following terms were used: “flexible sigmoidoscopy screening” and “colorectal cancer” and “relative risk” and “case-control studies OR cohort studies OR randomized controlled studies” (the detailed search strategy is available in **Supplementary Tables 1**–**3**).

Irrelevant studies were basically eliminated by selecting the titles and abstracts of two reviewers (LC and XS). For the remaining studies, a full-text review was conducted. Additionally, a review of the references in the identified articles was also carried out. When a disagreement appeared, an arbitrator (JL) was invited to resolve it.

### 2.2 Inclusion and exclusion criteria

Those studies that met the following PICO(S) criteria (participants, interventions, comparators, outcomes, study designs) were included:

#### 2.2.1 Participants

Individuals who were older than or equal to 18 years and had not been diagnosed with CRC.

#### 2.2.2 Interventions

At least once FS was conducted, including mass screening, opportunistic screening, whether or not to enter surveillance.

#### 2.2.3 Comparators

The control groups mainly included a non-screening method or other screening methods [like fecal immunochemical tests (FITs)].

#### 2.2.4 Outcomes

Incidence and mortality of CRC.

#### 2.2.5 Study designs

Cohort studies, retrospective cohort studies, case–control studies, and randomized controlled studies.

The excluded criteria were as follows:

1) FS screening studies were conducted only in patients with precancerous lesions, colonic melanosis, *Helicobacter pylori* infection, and enterotoxigenic *Bacteroides fragilis* (ETBF) coexisting with pks^+^ *Escherichia coli.*
2) Study designs that were based on surveillance only.
3) Publications in duplicate.
4) The studies involved conference abstracts, letters, expert opinions, case reports, and reviews.
5) Non-English language.

### 2.3 Data extraction and quality assessment

The following information was obtained from the publications: first author name, date of publication, study design, country, intervention, comparison, study period, sample size, frequency and period of FS screening, enrollment age, gender, follow-up time, adjustment/matching, and outcomes. Missing or incomplete data of the included studies were tried to be found from the corresponding authors if necessary and feasible.

The Newcastle-Ottawa quality assessment scale (NOS) and the *Jadad* scale were applied to assess the quality of the included studies (33), and the “Grading of Recommendations Assessment, Development and Evaluation System” (GRADE) was used to classify the quality of evidence.

### 2.4 Primary outcomes

Mortality and incidence of CRC after FS screening were selected as the primary outcomes.

### 2.5 Secondary outcomes

The subgroups were divided from the mortality and incidence of CRC that received FS according to different comparisons, interventions, sexes, ages, stages, and countries.

### 2.6 Statistical analysis

The relative risk (RR) was applied as an estimate of the effect of interest. Random-effects models were used to calculate study-specific RR estimates that considered both within-study and between-study variations. Heterogeneity analysis was performed using the chi-square test and *I* ^2^. “*P* < 0.1 and *I* ^2^ > 50%” indicated a significant heterogeneity. Sensitivity analysis was performed to evaluate the robustness of the results by sequentially omitting each study. Funnel plots were used to assess publication bias if 10 or more studies were included. *RevMan* was used to calculate all statistical analyses. *P*-values with two-tailed less than 0.05 were considered statistically significant, except for heterogeneity.

## 3 Results

### 3.1 Literature search

**Figure 1:**
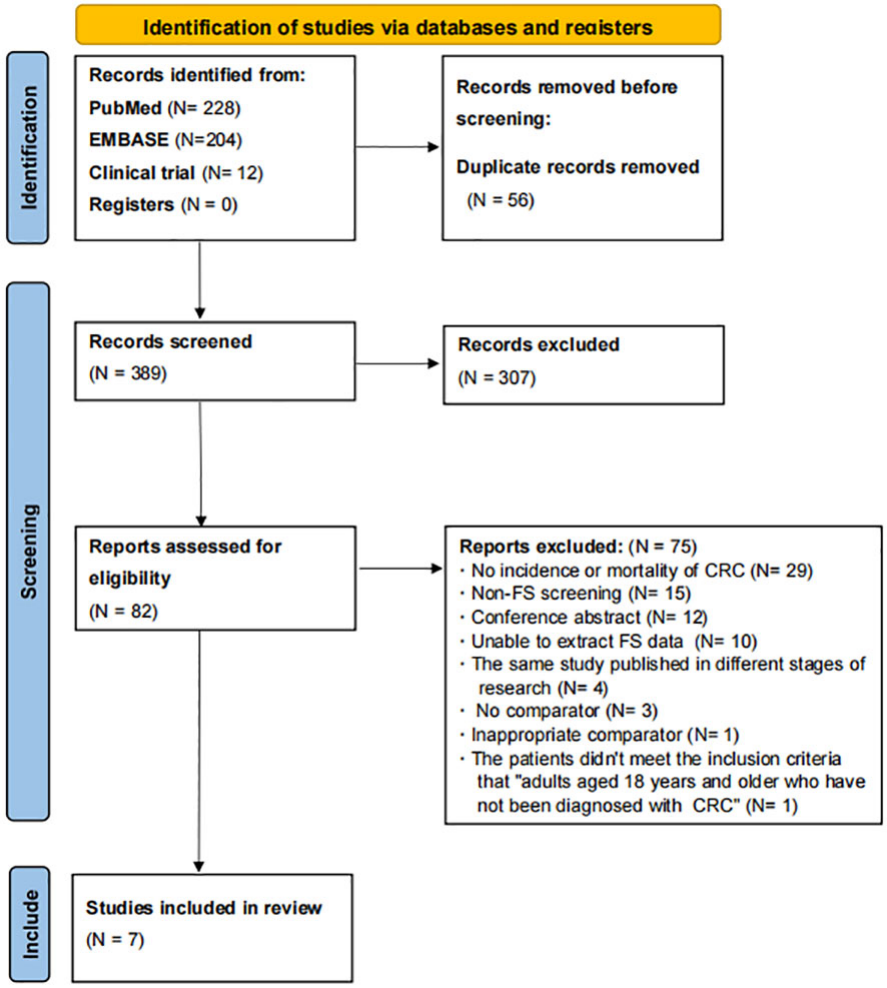
PRISMA flowchart of literature search and study selection.

A total of 444 articles were found according to the PubMed, EMBASE, and ClinicalTrials.gov search strategies, as shown in **Figure 1**. After deleting duplicate articles, there were 388 articles left. As a result of reviewing the titles and abstracts, 307 non-compliant articles were excluded and 1 potentially compliant article from the reference selection was included, leaving 82 articles. Then, 75 articles were excluded for the following reasons: no incidence or mortality of CRC (*N* = 29), non-FS screening (*N* = 15), conference abstract (*N* = 12), unable to extract FS data (*N* = 10), the same study published in different stages of research (*N* = 4), no comparator (*N* = 3), inappropriate comparator (*N* = 1), and the patients did not meet the inclusion criteria “adults aged 18 years and older who have not been diagnosed with CRC” (*N* = 1) (**Supplementary Table 4**). In the end, we included seven articles from PubMed, EMBASE, and ClinicalTrials.gov.

### 3.2 Study characteristics and quality assessment

There were six RCTs (25–28, 30, 31) and one cohort study (29), among which four studies enrolled more than 100,000 participants, two included 10,000 to 100,000 participants, and one enrolled 100 to 1,000 participants. In the six RCTs, a total of four studies used FS for screening (26–28, 31), and two used FS followed by surveillance (25, 30). Regarding the FS comparator, one study used FIT (30), four did not screen (26–28, 31), and one used usual care (25). Participants were mainly from Norway (three studies) (28, 30, 31), Italy (one study) (27), the United States (one study) (25), and the United Kingdom (one study) (26). Both incidence and mortality of CRC were investigated together by five studies, while a single study reported only incidence (**Tables 1**, **2**).

**Table 1:** Characteristics of studies included in the meta-analysis.

Study	Sample size (I/C)	Sex (M/F)		Age (years)	Design	Country	Study period	Follow-up (years)		Adjustments or match	Study quality	
		I	C					Incidence	Mortality		NOS	Jadad
Senore et al., 2022 (27)	27,047 (9,911/17,136)	NR	NR	55–64	RCT	Italy	1995–2016	Median: 15.4 (14.8–16.1)	Median: 18.8 (17.0–19.6)	Sex, age, trial center, and rate differences with 95% CIs	–	3
Miller et al., 2019 (25)	154,887 (77,443/77,444)	38,340/39,103	38,338/39,106	55–74	RCT	USA	1993–2015	Median: 15.8 (13.2–18.0)	Median: 16.8 (14.4–18.9)	–	–	3
Holme et al., 2018 (28)	88,397 (10,271/78,126)	10,255/38,872	10,297/39,254	50–64	RCT	Norway	1999–2015	Median: 14.8		Age	–	3
Atkin et al., 2017 (26)	153,557 (40,621/112,936)	20,489/20,132	55,339/57,597	55–64	RCT	UK	1994–2014	Median: 17.1		Non-compliance with screening	–	3
Wu et al., 2014 (29)	138,297 (95,359/42,938)	47,232/48,127	24,432/18,506	50–75	CS	USA	2000–2010	11	–	Age, sex, race/ethnicity, smoking status, CCIS, and comorbidity	8	–
Thiis-Evensen et al., 2013 (31)	799 (400/399)	NR	NR	50–59	RCT	Norway	1983–2008	26		–	–	2
Randel et al., 2021 (30)	139,291 (69,195/70,096)	34,068/35,127	34,601/35,495	50–74	RCT	Norway	2012–2019	8		Age	–	3

RCT, randomized controlled trial; CS, cohort study; Y, year; I, intervention; C, control; M, male; F, female; CCIS, Charlson comorbidity index score.

**Table 2:** Characteristics of the interventions in the included studies.

Authors	Intervention	Comparator	Numbers of outcomes		Frequency	Timing
			Diagnosed (I/C)	Deaths (I/C)		
Senore et al., 2022 (27)	FS	Never screened	468/184	56/157	1 time	Before diagnosis
Miller et al., 2019 (25)	FS	Usual care^a^	1,461/1,761	417/549	3 times	1993–2015
Holme et al., 2018 (28)	FS	Never screened	189/1,751	64/530	1 time	1999–2001
Atkin et al., 2017 (26)	FS	Never screened	776/3,253	215/996	1 time	1994–1999
Wu et al., 2014 (29)	FS	Colonoscopy screening	214/27	–	1 time	2000–2010
Thiis-Evensen et al., 2013 (31)	FS	Never screened	7/19	1/7	1 time	1983
Randel et al., 2021 (30)	FS	FIT	202/260	–	1 time	2012–2019

a Participants in the usual care arm could be screened under the care of their physician.

FIT, fecal immunochemical testing.

The quality assessment is displayed in **Table 1** (**Supplementary Tables 5**, **6**). In the cohort study (29), eight stars were received in NOS (10 as a full score). While in RCTs, five (25–28, 30) of the six studies received three stars (five as a full score), and one (31) received two stars on the Jadad scale because no blinding method was used. GRADE was applied to assess the quality of evidence. Both incidence and mortality in the RCTs were rated as “high” certainty. Meanwhile, the incidence in the cohort study was assessed as a “moderate” certainty (as the observational study, it had a rigorous methodology and the RR was greater than 2) (see **Supplementary Table 7** for more details).

### 3.3 Primary outcomes

**Figure 2:**
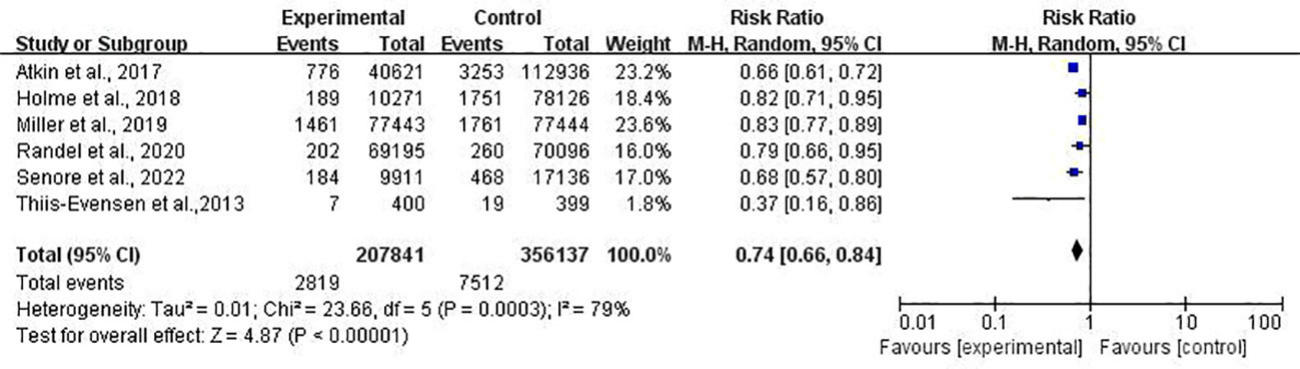
Forest plot of CRC incidence after FS.

In the incidence meta-analysis, combined data from six studies (25–28, 30, 31) covering 563,978 individuals were analyzed. The results showed that FS caused a 26% reduction in the incidence of CRC (RR, 0.74; 95% CI, 0.66–0.84) (**Figure 2**).

**Figure 3:**
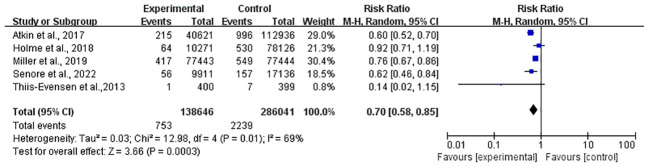
Forest plot of CRC mortality after FS.

Meanwhile, a total of 424,687 individuals from five studies (25–28, 31) were included in the mortality meta-analysis, which showed a 30% reduction (RR, 0.70; 95% CI, 0.88–0.85) (**Figure 3**).

However, significant heterogeneity was found both in the incidence (*I* ^2^ = 79%, *P* < 0.01) and mortality (*I* ^2^ = 69%, *P* = 0.01) studies, and a subgroup analysis was scheduled in the next section. In the sensitivity analysis, the significance of the results did not change after removing any study in both incidence and mortality. In light of the limited number of included incidence (25–28, 30, 31) and mortality (25–28, 31) studies (only six and five studies, respectively), the funnel plot asymmetry test was not performed.

### 3.4 Secondary outcomes

Secondary outcomes mainly included subgroups from incidence and mortality of CRC, such as comparator, intervention, sex, tumor site, stage of CRC, age, and country (**Tables 3**, **4**).

**Table 3:** Subgroup analysis of CRC incidence reduction after FS.

Subgroups	Number of studies	Pooled RR (95% CI)	*Z*	*P*	Heterogeneity	
					*I* ^2^ (%)	*P_h_*
Comparator						
Never screened (26–28, 31)	4	0.70 (0.61–0.81)	4.94	<0.00001	64	0.04
Usual care (25)	1	0.83 (0.77–0.89)	5.33	<0.00001	NA	NA
FIT (30)	1	0.79 (0.66–0.95)	2.56	0.01	NA	NA
Intervention						
Screening (26–28, 31)	4	0.70 (0.61–0.81)	4.94	<0.00001	64	0.04
Screening followed by surveillance (25, 30)	2	0.82 (0.77–0.88)	5.89	<0.00001	0	0.60
Sex						
Male (25–27, 30)	4	0.71 (0.63–0.82)	4.91	<0.00001	72	0.01
Female (25–27, 30)	4	0.77 (0.66–0.91)	3.19	0.001	70	0.02
Site						
Proximal (25–27, 30)	4	0.80 (0.63–1.01)	1.85	0.06	88	<0.0001
Distal (25–27, 30)	4	0.64 (0.46–0.88)	2.69	0.007	95	<0.00001
Stage						
Stage I (25, 30)	2	0.88 (0.75–1.03)	1.61	0.11	34	0.22
Stage II (25, 30)	2	0.61 (0.31–1.19)	1.45	0.15	85	0.009
Stage III (25, 30)	2	0.78 (0.68–0.90)	3.52	0.0004	0	0.68
Stage IV (25, 30)	2	0.72 (0.60–0.86)	3.57	0.0004	0	0.63
Age						
55–59 years (26, 27)	2	0.66 (0.59–0.73)	7.63	<0.00001	0	0.90
≥60 years (26, 27)	2	0.67 (0.61–0.74)	8.28	<0.00001	0	0.80
Country						
Norway (28, 30, 31)	3	0.78 (0.66–0.93)	2.80	0.005	40	0.19
Italy (27)	1	0.68 (0.57–0.80)	4.48	<0.00001	NA	NA
USA (25)	1	0.83 (0.77–0.89)	5.33	<0.00001	NA	NA
UK (26)	1	0.66 (0.61–0.72)	10.39	<0.00001	NA	NA

NA, not available.

**Table 4:** Subgroup analysis of CRC mortality reduction after FS.

Subgroups	Number of studies	Pooled RR (95% CI)	*Z*	*P*	Heterogeneity	
					*I* ^2^ (%)	*P_h_*
Comparator						
Never screened (26–28, 31)	4	0.67 (0.51–0.89)	2.73	0.006	70	0.02
Usual care (25)	1	0.76 (0.67–0.86)	4.25	<0.0001	NA	NA
Intervention						
Screening (26–28, 31)	4	0.67 (0.51–0.89)	2.73	0.006	70	0.02
Screening followed by surveillance (25)	1	0.76 (0.67–0.86)	4.25	<0.0001	NA	NA
Sex						
Male (25–27)	3	0.62 (0.54–0.71)	6.73	<0.00001	19	0.29
Female (25–27)	3	0.76 (0.61, 0.94)	2.49	0.01	47	0.15
Site						
Proximal (25–27)	3	0.94 (0.83–1.07)	0.91	0.36	0	0.93
Distal (25–27)	3	0.40 (0.29–0.56)	5.48	<0.00001	72	0.03
Age						
55–59 years (26, 27)	2	0.52 (0.42–0.64)	5.97	<0.00001	0	0.67
≥60 years (26, 27)	2	0.67 (0.56–0.79)	4.71	<0.00001	0	0.95
Country						
Norway (28, 31)	2	0.49 (0.09–2.77)	0.81	0.42	67	0.08
Italy (27)	1	0.62 (0.46–0.84)	3.12	0.002	NA	NA
USA (25)	1	0.76 (0.67–0.86)	4.25	<0.0001	NA	NA
UK (26)	1	0.60 (0.52–0.70)	6.81	<0.00001	NA	NA

NA, not available.

#### 3.4.1 Subgroups of CRC incidence

##### 3.4.1.1 Comparator

FS had a more protective effect on the incidence of CRC than never screened (26–28, 31) (RR, 0.70; 95% CI, 0.61–0.81), usual care (25) (RR, 0.83; 95% CI, 0.77–0.89), and FIT (30) (RR, 0.79; 95% CI, 0.66–0.95).

##### 3.4.1.2 Intervention

A reduction in CRC incidence was observed both in the FS screening group (26–28, 31) (RR, 0.70; 95% CI, 0.61–0.81) and in the screening followed by surveillance group (25, 30) (RR, 0.82; 95% CI, 0.77–0.88).

##### 3.4.1.3 Sex

Both men (25–27, 30) (RR, 0.71; 95% CI, 0.63–0.82) and women (25–27, 30) (RR, 0.77; 95% CI, 0.66–0.91) who received FS were reported to have a significant reduction in the incidence of CRC.

##### 3.4.1.4 Tumor site

At the distal site of CRC (25–27, 30), FS had a beneficial effect on incidence (RR, 0.64; 95% CI, 0.46–0.88) but was not reported at the proximal site (25–27, 30) (RR, 0.80; 95% CI, 0.63–1.01).

##### 3.4.1.5 Stage of CRC

FS could reduce the CRC incidence of stage III (25, 30) (RR, 0.78; 95% CI, 0.68–0.90) and stage IV (25, 30) (RR, 0.72; 95% CI, 0.60–0.86), but not in stage I (25, 30) (RR, 0.88; 95% CI, 0.75–1.03) and stage II (25, 30) (RR, 0.61; 95% CI, 0.31–1.19).

##### 3.4.1.6 Age

Individuals who were 55 to 59 years old (26, 27) (RR, 0.66; 95% CI, 0.99–0.73) and older than 60 years of age (26, 27) (RR, 0.67; 95% CI, 0.61–0.74) who received FS had a reduction in the incidence of CRC.

##### 3.4.1.7 Country

FS could decrease CRC incidence in people who came from Norway (28, 30, 31) (RR, 0.78; 95% CI, 0.66–0.93), Italy (27) (RR, 0.68; 95% CI, 0.57–0.80), the United States (25) (RR, 0.83; 95% CI, 0.77–0.89), and the United Kingdom (26) (RR, 0.66; 95% CI, 0.61–0.72).

#### 3.4.2 Subgroups of CRC mortality

##### 3.4.2.1 Comparator

FS showed a more effective reduction in CRC mortality compared with never screened (26–28, 31) (RR, 0.67; 95% CI, 0.51–0.89) and usual care (25) (RR, 0.76; 95% CI, 0.67–0.86).

##### 3.4.2.2 Intervention

Reductions were observed both in the FS screening group (26–28, 31) (RR, 0.67; 95% CI, 0.51–0.89) and the screening followed by surveillance group (25) (RR, 0.76; 95% CI, 0.67–0.86) in CRC mortality.

##### 3.4.2.3 Sex

CRC mortality was reduced in men (25–27) (RR, 0.62; 95% CI, 0.54–0.71) and women (25–27) (RR, 0.76; 95% CI, 0.61–0.94) who received FS.

##### 3.4.2.4 Tumor site

FS could reduce the mortality of distal CRC (25–27) (RR, 0.40; 95% CI, 0.29–0.56) but not in the proximal area (25–27) (RR, 0.94; 95% CI, 0.83–1.07).

##### 3.4.2.5 Age

FS could decrease CRC mortality in people aged 55 to 59 years (26, 27) (RR, 0.52; 95% CI, 0.42–0.64) and older than 60 years (26, 27) (RR, 0.67; 95% CI, 0.56–0.79).

##### 3.4.2.6 Country

FS was observed as a positive protection against CRC mortality in people from Italy (27) (RR, 0.62; 95% CI, 0.46–0.84), the United States (25) (RR, 0.76; 95% CI, 0.67–0.86), and the United Kingdom (26) (RR, 0.60; 95% CI, 0.52–0.70), but not from Norway (28, 31) (RR, 0.49; 95% CI, 0.09–2.77).

## 4 Discussion

RCTs, considered the gold standard for assessing the effectiveness of screening, can reduce potential bias in their design and conduct and, at the same time, balance potential confounders such as self-selection and recall bias. This project also includes observational studies (such as cohort studies), which are also considered reliable sources of evidence. In particular, there is a difference in purpose between RCT screening analyses and observational studies (34): the RCT screening analysis estimates the impact of providing screening (ignoring the actual use), while the observational study estimates the impact of the actual application of screening. Our study finally included six RCTs and one CS. Due to the fact that only one study from a single type of study cannot be analyzed in the meta-analysis, all data applied in this meta-analysis came from RCTs.

This meta-analysis suggests that the receiving of FS could reduce the incidence and mortality of CRC by 26% and 30%, respectively. According to the analysis of the incidence and mortality subgroups, FS showed a significant protective effect in men, women, the distal site of CRC, stages III to IV (only in incidence), and people 55 to 59 years and over 60 years old, but it was not observed at the proximal site, stages I to II (only in incidence), and people from Norway (only in mortality). The potential reasons for these negative results are as follows:

### 4.1 The potential reasons for the negative results that FS could not reduce the incidence and mortality of CRC at the proximal site

Advanced age (older than or equal to 50 years) as a risk factor could increase the incidence of proximal CRC (7, 35, 36), and polyps in the proximal colon are more likely to progress to CRC, which results in the incidence of proximal CRC to be high (37, 38). Furthermore, the detectable length of FS is within 60 cm from the anus, leading to approximately 50% tumor and 34% polyps beyond the scope of the FS examination (39, 40). Therefore, CRC at the proximal site seems to be impossible to be reduced by FS in incidence and mortality. However, we still performed a subgroup analysis of tumor location that was also observed in the original studies included in this meta-analysis. This may be mainly due to the fact that FS is a preliminary screening and that FS-positive participants will undergo a routine colonoscopy, which leads to indirect monitoring of the right colon. However, the sad truth is that this meta-analysis did not observe that FS has a protective effect on the incidence and mortality of proximal CRC.

### 4.2 The potential reasons for the negative results that FS could not reduce the incidence of CRC in stages I and II

The vulnerable site of CRC was found to shift from left to right with age (41, 42). That is, more and more cases of CRC occurred in the right half of the colon. The FS examination is relatively simple and inexpensive compared with colonoscopy, while the disadvantage of FS is that it is not possible to examine all the colon (especially the right colon), leading to most CRCs located in the right colon that cannot be prevented at an early stage. Furthermore, the lack of typical symptoms in the early stage of CRC also contributes to the fact that CRC in stages I and II cannot be observed.

### 4.3 The potential reasons for the negative results that FS could not reduce the CRC mortality of people from Norway

Norwegian women ranked first in the incidence rate of CRC (1, 31). Compared with distal colon cancer, proximal colon cancer shows more invasion and a poor prognosis (43). According to studies, women are at a higher risk of developing proximal colon cancer than men, which can further affect the effectiveness of FS screening in women. Furthermore, some studies have confirmed that FS screening in Norway can reduce the mortality of CRC in men, but it has little impact on women (28, 44). Therefore, compared with FS examination, a thorough mucosal examination (such as colonoscopy) may be a better option for women with a high incidence of CRC in Norway.

Although our research has produced positive results, it still has some limitations: 1) Publication bias and lead time bias may lead to an overestimation of screening effects. 2) Since only English-language studies were included, qualified articles in other languages might have been overlooked. 3) It is impossible to completely exclude confounder factors from the results of this study. For example, infection with specific bacterial species, such as *H. pylori*, *Clostridium*, and enterotoxigenic *B. fragilis*, can increase the risk of CRC (45–47). 4) The included studies are mainly from countries in Europe and North America, so the results may not be generalizable to populations elsewhere. 5) As the heterogeneity of this meta-analysis was high, we tried to explore the source of heterogeneity from the subgroup analysis. Our study evaluated heterogeneity by dividing the interventions into two groups: screening followed by surveillance and screening for FS. It was observed that heterogeneity was markedly reduced in the screening group followed by surveillance (*I* ^2^ = 0%). However, a significant heterogeneity was still observed in the FS screening group (*I* ^2^ = 64%). 6) According to the CRC statistics released in 2023 (48), a marked increase in young and middle-aged adults and a decrease in elderly people make the population of CRC patients rapidly younger. Between 2011 and 2019, the incidence rate in people under 50 years of age and 50–54 years of age increased by 1.9% per year, and the proportion of newly diagnosed people under 55 years of age nearly doubled, from 11% in 1995 to 20% in 2019. Therefore, it is necessary to clarify the application, clinical importance, and potential efficacy of FS in the diagnosis of tumors in the younger population (<50 years). Unfortunately, the age groups were only divided into two sections (55–59 years and older than 60 years) of the original studies included in this meta-analysis; therefore, the younger population (<50 years) cannot be observed as expected. Although the specific effect on the younger age group (<50 years) is unknown, combined with the epidemiological background of CRC being gradually “younger,” and FS capable of reducing the incidence and mortality of CRC, young people should also pay attention to CRC detection, as recommended by the US Preventive Services Task Force (USPSTF) to detect CRC in adults 45 to 49 years of age (recommendation B) (49).

## 5 Conclusion

In conclusion, our analysis shows that current FS could reduce the incidence and mortality of CRC. Distal sites, older than 55 years, and stages III to IV of CRC appear to be protected more effectively by FS. This meta-analysis may have implications in the revision of current CRC guidelines, especially in those countries where organized screening is lacking and opportunistic colonoscopy has challenges in terms of uptake, resources, and costs; that is, FS can be proposed as a first-stage screening or in combination with fecal immunochemical testing in settings with opportunistic CRC screening. Furthermore, it may provide a more precise reference to the age range of “A recommendation” in the guidelines (the USPSTF has recommended that CRC screening be used for all adults 50 to 75 years of age as “A recommendation” in 2021) (13), because it found that FS can provide protective effects on the incidence and mortality of CRC at 55–59 years of age and older than 60 years. CRC has a wide range of health implications, which affect people’s quality of life and mortality. It is expected that FS will positively influence public health and that it should be encouraged around the world. To confirm this finding, further prospective clinical studies should be conducted based on a larger-scale population.

## Data availability statement

The raw data supporting the conclusions of this article will be made available by the authors, without undue reservation.

## Author contributions

XW: Conceptualization, Data curation, Formal analysis, Resources, Writing – original draft, Writing – review & editing. LC: Conceptualization, Data curation, Formal analysis, Resources, Writing – original draft, Writing – review & editing. XS: Validation, Writing – review & editing. GZ: Funding acquisition, Writing – review & editing. BN: Software, Writing – review & editing. XM: Project administration, Writing – review & editing. JL: Supervision, Visualization, Writing – review & editing.
