# Supplementary material for: Is flexible sigmoidoscopy screening associated with reducing colorectal cancer incidence and mortality? a meta-analysis and systematic review
Source: Front Oncol. 2023 Dec 13;13:1288086. doi: 10.3389/fonc.2023.1288086 (PMC10757863; doi:10.3389/fonc.2023.1288086)
Supplement: Supplementary file 1 [file Table_1.docx]

**Supplementary Table 1. Search Strategy Used in PubMed, December 11, 2022**

| **Number** | **Search Items** | **Items Found** |
| --- | --- | --- |
| 1 | (((flexible sigmoidoscopy screening[MeSH Terms]) OR flexible sigmoidoscopy screening) OR sigmoidoscopy screening[MeSH Terms]) OR sigmoidoscopy screening | 5,281 |
| 2 | ((((((((((((((((((((((((((“colorectal cancer”[MeSH Terms]) OR “colorectal cancer”[Title/Abstract]) OR “colorectal neoplasms”[MeSH Terms]) OR “colorectal neoplasms”[Title/Abstract]) OR “colorectal tumor”[MeSH Terms]) OR “colorectal tumor”[Title/Abstract]) OR “colon and rectal cancer”[Title/Abstract]) OR “colon and rectal tumor”[Title/Abstract]) OR “colon and rectal neoplasm”[Title/Abstract]) OR “colon and rectum cancer”[Title/Abstract]) OR “colon and rectum tumor”[Title/Abstract]) OR “colon and rectum neoplasm”[Title/Abstract]) OR “colon cancer and rectal cancer”[Title/Abstract]) OR “colon cancer and rectal neoplasm”[Title/Abstract]) OR “colon cancer and rectum neoplasm”[Title/Abstract]) OR “colon cancer and rectal tumor”[Title/Abstract]) OR “colon cancer and rectum cancer”[Title/Abstract]) OR “colonic neoplasm and rectal cancer”[Title/Abstract]) OR “colonic neoplasm and rectal neoplasm”[Title/Abstract]) OR “colonic neoplasm and rectum neoplasm”[Title/Abstract]) OR “colonic neoplasm and rectal tumor”[Title/Abstract]) OR “colonic neoplasm and rectum cancer”[Title/Abstract]) OR “colon tumor and rectal cancer”[Title/Abstract]) OR “colon tumor and rectal neoplasm”[Title/Abstract]) OR “colon tumor and rectum neoplasm”[Title/Abstract]) OR “colon tumor and rectal tumor”[Title/Abstract]) OR “colon tumor and rectum cancer”[Title/Abstract] | 266,207 |
| 3 | (((((“relative risk”) OR “odds ratio”) OR “rate ratio”) OR “risk ratio”) OR “hazard ratio”) OR ratio | 1,660,599 |
| 4 | (((((((“case-control studies” [MeSH Terms]) OR “cohort studies” [MeSH Terms]) OR “randomized controlled studies” [MeSH Terms]) OR “randomised controlled studies” [MeSH Terms]) OR “cohort”) OR “case control”) OR “randomize control”) OR randomise control | 3,371,532 |
| 5 | #1 AND #2 AND #3 AND #4 | 228 |
