# Supplementary material for: Is flexible sigmoidoscopy screening associated with reducing colorectal cancer incidence and mortality? a meta-analysis and systematic review
Source: Front Oncol. 2023 Dec 13;13:1288086. doi: 10.3389/fonc.2023.1288086 (PMC10757863; doi:10.3389/fonc.2023.1288086)
Supplement: Supplementary file 2 [file Table_2.docx]

**Supplementary Table 2. Search Strategy Used in EMBASE, December 11, 2022**

| **Number** | **Search Items** | **Items Found** |
| --- | --- | --- |
| 1 | flexible sigmoidoscopy screening'/exp OR 'sigmoidoscopy screening'/exp OR flexible sigmoidoscopy screening OR sigmoidoscopy screening | 5,032 |
| 2 | colorectal cancer':ti,ab,kw OR 'colorectal neoplasm':ti,ab,kw OR 'colorectal tumor':ti,ab,kw OR 'colon and rectal cancer':ti,ab,kw OR 'colon and rectal tumor':ti,ab,kw OR 'colon and rectal neoplasm':ti,ab,kw OR 'colon and rectum cancer':ti,ab,kw OR 'colon and rectum tumor':ti,ab,kw OR 'colon and rectum neoplasm':ti,ab,kw OR 'colon cancer and rectal cancer':ti,ab,kw OR 'colon cancer and rectal neoplasm':ti,ab,kw OR 'colon cancer and rectum neoplasm':ti,ab,kw OR 'colon cancer and rectal tumor':ti,ab,kw OR 'colon cancer and rectum cancer':ti,ab,kw OR 'colonic neoplasm and rectal cancer':ti,ab,kw OR 'colonic neoplasm and rectal neoplasm':ti,ab,kw OR 'colonic neoplasm and rectum neoplasm':ti,ab,kw OR 'colonic neoplasm and rectal tumor':ti,ab,kw OR 'colonic neoplasm and rectum cancer':ti,ab,kw OR 'colon tumor and rectal cancer':ti,ab,kw OR 'colon tumor and rectal neoplasm':ti,ab,kw OR 'colon tumor and rectum neoplasm':ti,ab,kw OR 'colon tumor and rectal tumor':ti,ab,kw OR 'colon tumor and rectum cancer':ti,ab,kw | 191,424 |
| 3 | risk factor' OR 'relative risks' OR 'odds ratio' OR 'odds ratios' OR 'rate ratio' OR 'rate ratios' OR 'risk ratio' OR 'risk ratios' OR 'hazard ratio' OR 'hazard ratios' OR ratio | 3,187,337 |
| 4 | cohort study'/exp OR 'case control study'/exp OR 'randomized controlled study'/exp OR 'randomised controlled study'/exp OR 'case control' OR 'randomize control' OR 'randomise control' OR cohort | 2,392,829 |
| 5 | #1 AND #2 AND #3 AND #4 | 204 |
