# Supplementary material for: Is flexible sigmoidoscopy screening associated with reducing colorectal cancer incidence and mortality? a meta-analysis and systematic review
Source: Front Oncol. 2023 Dec 13;13:1288086. doi: 10.3389/fonc.2023.1288086 (PMC10757863; doi:10.3389/fonc.2023.1288086)
Supplement: Supplementary file 3 [file Table_3.docx]

**Supplementary Table 3. Search Strategy Used in Clinical trial, December 11, 2022**

| **Number** | **Search Items** | **Items Found** |
| --- | --- | --- |
| 1 | Condition or disease: colorectal cancer OR colorectal neoplasms OR colorectal tumor OR colon and rectal cancer OR colon and rectal tumor OR colon and rectal neoplasm OR colon and rectum cancer OR colon and rectum tumor OR colon and rectum neoplasm OR colon cancer and rectal cancer OR colon cancer and rectal neoplasm OR colon cancer and rectum neoplasm OR colon cancer and rectal tumor OR colon cancer and rectum cancer OR colonic neoplasm and rectal cancer OR colonic neoplasm and rectal neoplasm OR colonic neoplasm and rectum neoplasm OR colonic neoplasm and rectal tumor OR colonic neoplasm and rectum cancer OR colon tumor and rectal cancer OR colon tumor and rectal neoplasm OR colon tumor and rectum neoplasm OR colon tumor and rectal tumor OR colon tumor and rectum cancer | 7,352 |
| 2 | Other terms: flexible sigmoidoscopy screening OR sigmoidoscopy screening | 75 |
| 3 | Study Results: Studies with results | 56,301 |
| 4 | #1 AND #2 AND #3 | 12 |
