# Supplementary material for: Is flexible sigmoidoscopy screening associated with reducing colorectal cancer incidence and mortality? a meta-analysis and systematic review
Source: Front Oncol. 2023 Dec 13;13:1288086. doi: 10.3389/fonc.2023.1288086 (PMC10757863; doi:10.3389/fonc.2023.1288086)
Supplement: Supplementary file 4 [file Table_4.docx]

**Supplementary Table 4. Characteristics of Excluded Studies Involving Colorectal Cancer**

| **Database** | **Study** | **Reason for Exclusion** |
| --- | --- | --- |
| Clinical trial | Christine D Berg *et al*., 2022^1^ | Data of incidence/mortality of CRC were not reported |
|  | Joan M. Griffin *et al*., 2020^2^ | Data of incidence/mortality of CRC were not reported |
|  | Kenzie Cameron *et al*., 2011^3^ | Data of incidence/mortality of CRC were not reported |
|  | Kenzie A Cameron *et al*., 2011^4^ | Data of incidence/mortality of CRC were not reported |
|  | Bechara N Choucair *et al*., 2014^5^ | Data of incidence/mortality of CRC were not reported |
|  | Gloria Coronado *et al*., 2019^6^ | Data of incidence/mortality of CRC were not reported |
| Pubmed | *Wenjie Ma* *et al*., 2022^7^ | Unable to extract FS data |
|  | Amy B Knudsen *et al*., 2021^8^ | Outcomes were not involved in incidence/mortality of CRC |
|  | Martin C S Wong *et al*., 2019^9^ | Outcomes were not involved in incidence/mortality of CRC |
|  | *Wenjie Ma* *et al*., 2021^10^ | Unable to extract FS data |
|  | *Feng Guo* *et al*., 2021^11^ | Data of incidence/mortality of CRC associated with FS could not be obtained |
|  | Doubeni, C A *et al*., 2020^12^ | Inappropriate comparator |
|  | *Xiaosheng He* *et al*., 2019^13^ | There was no control group. Although FS screening was used, the data of FS were unable to be extracted |
|  | Warren, Andersen S *et al*., 2019^14^ | This was a study without FS screening |
|  | Carlos, C A *et al*., 2017^15^ | No comparator |
|  | Senore, C *et al*., 2014^16^ | Data of incidence/mortality of CRC were not reported |
|  | Kahi, C J *et al*., 2014^17^ | Although FS screening was used, rates of incidence/mortality could not be obtained |
|  | Doubeni, C A *et al*., 2013^18^ | This was a study without FS screening |
|  | Blom, J *et al*., 2008^19^ | This was a study without FS screening |
|  | Maw-Soan Soon *et al*., 2005^20^ | This was a study without FS screening |
|  | Michelle Cotterchio *et al*., 2005^21^ | Although FS screening was used, data of incidence/mortality of CRC were not reported |
|  | G Gondal *et al*., 2003^22^ | This was a study without FS screening |
|  | Newcomb, P A *et al*., 2003^23^ | This was a study without FS screening |
|  | H Brenner *et al*., 2001^24^ | This was a study without FS screening |
|  | M L Slattery *et al*., 2000^25^ | This was a study without FS screening |
|  | Sonnenberg, A *et al*., 2000^26^ | The specific cases of CRC could not be obtained |
|  | B C Tilley *et al*., 1999^27^ | Although FS screening was used, data of incidence/mortality of CRC were not reported |
|  | Kavanagh, A M *et al*., 1998^28^ | Unable to extract FS data |
|  | R Y Demers *et al*., 1994^29^ | Although FS screening was used, the data of FS were unable to be extracted |
|  | Selby, J V *et al*., 1992^30^ | This was a study without FS screening |
|  | Newcomb, P A *et al*., 1995^31^ | This was a study without FS screening |
|  | Knudsen, A B *et al*., 2012^32^ | This was a study without FS screening |
|  | Senore, C *et al*., 2019^33^ | This was a cost-effectiveness study which did not report data of incidence/mortality of CRC associated with FS |
|  | Ko, C W *et al*., 2019^34^ | The patients didn't meet the inclusion criteria that "adults aged 18 years and older who have not been diagnosed with CRC" |
|  | Click, B *et al*., 2018^35^ | Data of incidence/mortality of CRC were not reported |
|  | Doria-Rose, V P *et al*., 2014^36^ | No comparator |
|  | Nishihara, R *et al*., 2013^37^ | Data of screening group cannot be obtained |
|  | Segnan, N *et al*., 2011^38^ | This was a study without FS screening |
|  | Atkin WS *et al*., 2010^39^ | The same study published in different research stages as Atkin *et al*.,2017 |
|  | Steele, R. J. *et al*., 2020^40^ | Data of incidence/mortality of CRC associated with FS could not be obtained |
|  | Laiyemo, A. O. *et al*., 2010^41^ | Although FS screening was used, data of incidence/mortality of CRC were not reported |
|  | *Wang*, Y. R. *et al*., 2013^42^ | Data of incidence/mortality of CRC were not reported |
|  | W S Atkin *et al*., 2002^43^ | Although FS screening was used, data of incidence/mortality of CRC were not reported |
|  | Thiis-Evensen *et al*., 1999^44^ | The same study published in different research stages as Thiis-Evensen, E *et al*., 2013 |
|  | Schoen, R. E. *et al*., 2012^45^ | The same study published in different research stages as Miller *et al*., 2019 |
|  | Holme, Ø. *et al*., 2014^46^ | The same study published in different research stages as Holme *et al*., 2018 |
| Embase | *Yang,* K *et al*., 2022^47^ | Conference abstract |
|  | Zgraggen, A *et al*., 2022^48^ | Data of incidence/mortality of CRC were not reported, and the method of screening was colonoscopy rather than FS |
|  | *Wang*, K *et al*., 2021^49^ | Methods of screening of this study included colonoscopy and FS, but the data of FS were unable to be extracted |
|  | Laiyemo, A *et al*., 2021^50^ | Data of incidence/mortality of CRC were not reported |
|  | Abdel-Rahman, O *et al*., 2021^51^ | Data of incidence/mortality of CRC were not reported |
|  | Okereke, I *et al*., 2020^52^ | Conference abstract |
|  | Ezeofor, A *et al*., 2020^53^ | Conference abstract |
|  | Peterse, E F *et al*., 2018^54^ | Conference abstract |
|  | Peterse, E F P *et al*., 2018^55^ | Although FS screening was used, data of incidence/mortality of CRC were not reported |
|  | Nozaki, R *et al*., 2017^56^ | Conference abstract |
|  | Click, B H *et al*., 2017^57^ | Conference abstract |
|  | Lieberman, D *et al*., 2016^58^ | Data of incidence/mortality of CRC were not reported |
|  | Tang, R S *et al*., 2015^59^ | Although FS screening was used, data of incidence/mortality of CRC were not reported |
|  | Keller, S C *et al*., 2014^60^ | Conference abstract |
|  | Razzak, A *et al*., 2014^61^ | The aim of this study was to investigate the relationship between family history and the risk of CRC. Data of incidence/mortality of CRC were not reported |
|  | Mehta, S J *et al*., 2014^62^ | Although FS screening was used, data of incidence/mortality of CRC were not reported |
|  | Doubeni, C A *et al*., 2013^63^ | Although FS screening was used, data of incidence/mortality of CRC were not reported |
|  | *Wu*, B U *et al*., 2013^64^ | Conference abstract |
|  | Jeffers, K *et al*., 2012^65^ | Although FS screening was used, data of incidence/mortality of CRC were not reported |
|  | Courtney, R J *et al*., 2012^66^ | Although FS screening was used, data of incidence/mortality of CRC were not reported |
|  | Nishihara, R *et al*., 2012^67^ | Conference abstract |
|  | Doria-Rose, V P *et al*., 2011^68^ | Conference abstract |
|  | Burnet-Hartman, A *et al*., 2011^69^ | Data of incidence/mortality of CRC were not reported in patients with advanced adenoma |
|  | Aggarwal, A *et al*., 2011^70^ | This was a study without FS screening |
|  | Anand, N *et al*., 2009^71^ | This was a study without FS screening |
|  | Farraye, F A *et al*., 2004^72^ | Data of incidence/mortality of CRC were not reported |
|  | Holme, O. *et al*., 2013^73^ | Conference abstract |
|  | Atkin W.S. *et al*., 2010^74^ | Conference abstract |
|  | P A Newcomb *et al*., 1992^75^ | The screening method was rigid sigmoidoscopy or FS, but the data of FS were unable to be extracted |

**Reference:**

1. Berg CD. Screening for Colorectal Cancer in Older Patients (PLCO Screening Trial).

2. Griffin JM. Use of Telehealth In-home Messaging to Improve GI (Gastrointestinal) Endoscopy Completion Rates (GIVER).

3. Cameron K. Outreach for Patients That Are Newly Eligible for Colorectal Cancer Screening (UPQUAL).

4. Cameron KA. Outreach for Patients With Uncompleted Colorectal Cancer Screening Orders (UPQUAL).

5. Choucair BN. Effectiveness of Direct-to-Patient Outreach on Colorectal Cancer Screening Within a Low Income and Diverse Population.

6. Gloria Coronadom BG. Strategies and Opportunities to Stop Colon Cancer in Priority Populations (STOPCRC).

7. Ma W, Wang M, Wang K, Cao Y. Age at Initiation of Lower Gastrointestinal Endoscopy and Colorectal Cancer Risk Among US Women. *JAMA oncology*. Jul 1 2022;8(7):986-993. doi:10.1001/jamaoncol.2022.0883

8. Knudsen AB, Rutter CM, Peterse EFP, Lietz AP, Seguin CL, Meester RGS. Colorectal Cancer Screening: An Updated Modeling Study for the US Preventive Services Task Force. *JAMA*. May 18 2021;325(19):1998-2011. doi:10.1001/jama.2021.5746

9. Wong MCS, Rerknimitr R, Lee Goh K, Matsuda T, Kim HS, Wu DC. Development and Validation of the Asia-Pacific Proximal Colon Neoplasia Risk Score. *Clinical gastroenterology and hepatology : the official clinical practice journal of the American Gastroenterological Association*. Jan 2021;19(1):119-127 e1. doi:10.1016/j.cgh.2019.12.031

10. Ma W, Wang K, Nguyen LH, Joshi A, Cao Y. Association of Screening Lower Endoscopy With Colorectal Cancer Incidence and Mortality in Adults Older Than 75 Years. *JAMA oncology*. Jul 1 2021;7(7):985-992. doi:10.1001/jamaoncol.2021.1364

11. Guo F, Chen C, Holleczek B, Schottker B, Hoffmeister M, Brenner H. Strong Reduction of Colorectal Cancer Incidence and Mortality After Screening Colonoscopy: Prospective Cohort Study From Germany. *The American journal of gastroenterology*. May 1 2021;116(5):967-975. doi:10.14309/ajg.0000000000001146

12. Doubeni CA, Corley DA, Jensen CD, Schottinger JE, Lee JK. The effect of using fecal testing after a negative sigmoidoscopy on the risk of death from colorectal cancer. *Journal of medical screening*. Jun 2021;28(2):140-147. doi:10.1177/0969141320921427

13. He X, Hang D, Wu K, et al. Long-term Risk of Colorectal Cancer After Removal of Conventional Adenomas and Serrated Polyps. *Gastroenterology*. Mar 2020;158(4):852-861 e4. doi:10.1053/j.gastro.2019.06.039

14. Warren Andersen S, Blot WJ, Lipworth L, Steinwandel M, Murff HJ, Zheng W. Association of Race and Socioeconomic Status With Colorectal Cancer Screening, Colorectal Cancer Risk, and Mortality in Southern US Adults. *JAMA network open*. Dec 2 2019;2(12):e1917995. doi:10.1001/jamanetworkopen.2019.17995

15. Carlos CA, McCulloch CE, Hsu CY, Grimes B. Colon Cancer Screening among Patients Receiving Dialysis in the United States: Are We Choosing Wisely? *Journal of the American Society of Nephrology : JASN*. Aug 2017;28(8):2521-2528. doi:10.1681/ASN.2016091019

16. Senore C, Bonelli L, Sciallero S, Casella C, Santarelli A, Armaroli P. Assessing generalizability of the findings of sigmoidoscopy screening trials: the case of SCORE trial. *Journal of the National Cancer Institute*. Jan 2015;107(1):385. doi:10.1093/jnci/dju385

17. Kahi CJ, Myers LJ, Slaven JE, et al. Lower endoscopy reduces colorectal cancer incidence in older individuals. *Gastroenterology*. Mar 2014;146(3):718-725 e3. doi:10.1053/j.gastro.2013.11.050

18. Doubeni CA, Weinmann S, Adams K, Kamineni A, Buist DS, Ash AS. Screening colonoscopy and risk for incident late-stage colorectal cancer diagnosis in average-risk adults: a nested case-control study. *Annals of internal medicine*. Mar 5 2013;158(5 Pt 1):312-20. doi:10.7326/0003-4819-158-5-201303050-00003

19. Blom J, Yin L, Liden A, Dolk A, Jeppsson B, Pahlman L. A 9-year follow-up study of participants and nonparticipants in sigmoidoscopy screening: importance of self-selection. *Cancer epidemiology, biomarkers & prevention : a publication of the American Association for Cancer Research, cosponsored by the American Society of Preventive Oncology*. May 2008;17(5):1163-8. doi:10.1158/1055-9965.EPI-07-2764

20. Soon MS, Kozarek RA, Ayub K, Soon A, Lin TY, Lin OS. Screening colonoscopy in Chinese and Western patients: a comparative study. *The American journal of gastroenterology*. Dec 2005;100(12):2749-55. doi:10.1111/j.1572-0241.2005.00355.x

21. Cotterchio M, Manno M, Klar N, McLaughlin J, Gallinger S. Colorectal screening is associated with reduced colorectal cancer risk: a case-control study within the population-based Ontario Familial Colorectal Cancer Registry. *Cancer causes & control : CCC*. Sep 2005;16(7):865-75. doi:10.1007/s10552-005-2370-3

22. Gondal G, Grotmol T, Hofstad B, Bretthauer M, Eide TJ, Hoff G. Grading of distal colorectal adenomas as predictors for proximal colonic neoplasia and choice of endoscope in population screening: experience from the Norwegian Colorectal Cancer Prevention study (NORCCAP). *Gut*. Mar 2003;52(3):398-403. doi:10.1136/gut.52.3.398

23. Newcomb PA, Storer BE, Morimoto LM, Templeton A, Potter JD. Long-term efficacy of sigmoidoscopy in the reduction of colorectal cancer incidence. *Journal of the National Cancer Institute*. Apr 16 2003;95(8):622-5. doi:10.1093/jnci/95.8.622

24. Brenner H, Arndt V, Sturmer T, Stegmaier C, Ziegler H, Dhom G. Long-lasting reduction of risk of colorectal cancer following screening endoscopy. *British journal of cancer*. Sep 28 2001;85(7):972-6. doi:10.1054/bjoc.2001.2023

25. Slattery ML, Edwards SL, Ma KN, Friedman GD. Colon cancer screening, lifestyle, and risk of colon cancer. *Cancer causes & control : CCC*. Jul 2000;11(6):555-63. doi:10.1023/a:1008924115604

26. Sonnenberg A, Delco F, Inadomi JM. Cost-effectiveness of colonoscopy in screening for colorectal cancer. *Annals of internal medicine*. Oct 17 2000;133(8):573-84. doi:10.7326/0003-4819-133-8-200010170-00007

27. Tilley BC, Vernon SW, Myers R, et al. The Next Step Trial: impact of a worksite colorectal cancer screening promotion program. *Preventive medicine*. Mar 1999;28(3):276-83. doi:10.1006/pmed.1998.0427

28. Kavanagh AM, Giovannucci EL, Fuchs CS, Colditz GA. Screening endoscopy and risk of colorectal cancer in United States men. *Cancer causes & control : CCC*. Aug 1998;9(4):455-62. doi:10.1023/a:1008884021049

29. Demers RY, Parsons KC. Colorectal cancer incidence in pattern and model makers: evidence from a screening program. *American journal of industrial medicine*. Jul 1994;26(1):33-45. doi:10.1002/ajim.4700260104

30. Selby JV, Friedman GD, Quesenberry CP, Jr., Weiss NS. A case-control study of screening sigmoidoscopy and mortality from colorectal cancer. *The New England journal of medicine*. Mar 5 1992;326(10):653-7. doi:10.1056/NEJM199203053261001

31. Newcomb PA, Storer BE, Marcus PM. Cigarette smoking in relation to risk of large bowel cancer in women. *Cancer research*. Nov 1 1995;55(21):4906-9.

32. Knudsen AB, Hur C, Kuntz KM, Haug U, Gazelle GS. Effectiveness and cost-effectiveness of once-only screening for colorectal cancer with colonoscopy or computed tomographic colonography. Conference Abstract. *Gastroenterology*. 2012;142(5):S141-S142.

33. Senore C, Hassan C, Regge D, et al. Cost-effectiveness of colorectal cancer screening programmes using sigmoidoscopy and immunochemical faecal occult blood test. *Journal of medical screening*. Jun 2019;26(2):76-83. doi:10.1177/0969141318789710

34. Ko CW, Doria-Rose VP, Barrett MJ, Kamineni A, Enewold L, Weiss NS. Screening colonoscopy and flexible sigmoidoscopy for reduction of colorectal cancer incidence: A case-control study. *PLoS One*. 2019;14(12):e0226027. doi:10.1371/journal.pone.0226027

35. Click B, Pinsky PF, Hickey T, Doroudi M, Schoen RE. Association of Colonoscopy Adenoma Findings With Long-term Colorectal Cancer Incidence. *JAMA*. May 15 2018;319(19):2021-2031. doi:10.1001/jama.2018.5809

36. Doria-Rose VP, Levin TR, Palitz A, Conell C, Weiss NS. Ten-year incidence of colorectal cancer following a negative screening sigmoidoscopy: an update from the Colorectal Cancer Prevention (CoCaP) programme. *Gut*. Feb 2016;65(2):271-7. doi:10.1136/gutjnl-2014-307729

37. Nishihara R, Wu K, Lochhead P, Morikawa T, Liao X, Qian ZR. Long-term colorectal-cancer incidence and mortality after lower endoscopy. *The New England journal of medicine*. Sep 19 2013;369(12):1095-105. doi:10.1056/NEJMoa1301969

38. Segnan N, Armaroli P, Bonelli L, Risio M, Sciallero S. Once-only sigmoidoscopy in colorectal cancer screening: follow-up findings of the Italian Randomized Controlled Trial--SCORE. *Journal of the National Cancer Institute*. Sep 7 2011;103(17):1310-22. doi:10.1093/jnci/djr284

39. Atkin WS, Edwards R, Kralj-Hans I, Wooldrage K, Hart AR, Northover JM. Once-only flexible sigmoidoscopy screening in prevention of colorectal cancer: a multicentre randomised controlled trial. *Lancet (London, England)*. May 8 2010;375(9726):1624-33. doi:10.1016/S0140-6736(10)60551-X

40. Steele RJ, Carey FA, Stanners G, Lang J, Brand J. Randomized controlled trial: Flexible sigmoidoscopy as an adjunct to faecal occult blood testing in population screening. *Journal of medical screening*. Jun 2020;27(2):59-67. doi:10.1177/0969141319879955

41. Laiyemo AO, Doubeni C, Pinsky PF, Doria-Rose VP, Bresalier R, Lamerato LE. Race and colorectal cancer disparities: health-care utilization vs different cancer susceptibilities. *Journal of the National Cancer Institute*. Apr 21 2010;102(8):538-46. doi:10.1093/jnci/djq068

42. Wang YR, Cangemi JR, Loftus EV, Jr., Picco MF. Risk of colorectal cancer after colonoscopy compared with flexible sigmoidoscopy or no lower endoscopy among older patients in the United States, 1998-2005. *Mayo Clinic proceedings*. May 2013;88(5):464-70. doi:10.1016/j.mayocp.2012.12.012

43. Atkin WS, Cook CF, Cuzick J, Edwards R, Northover JM. Single flexible sigmoidoscopy screening to prevent colorectal cancer: baseline findings of a UK multicentre randomised trial. *Lancet (London, England)*. Apr 13 2002;359(9314):1291-300. doi:10.1016/S0140-6736(02)08268-5

44. Thiis-Evensen E, Hoff GS, Sauar J, Langmark F, Majak BM. Population-based surveillance by colonoscopy: effect on the incidence of colorectal cancer. Telemark Polyp Study I. *Scandinavian journal of gastroenterology*. Apr 1999;34(4):414-20. doi:10.1080/003655299750026443

45. Schoen RE, Pinsky PF, Weissfeld JL, Yokochi LA, Church T. Colorectal-cancer incidence and mortality with screening flexible sigmoidoscopy. *The New England journal of medicine*. Jun 21 2012;366(25):2345-57. doi:10.1056/NEJMoa1114635

46. Holme O, Loberg M, Kalager M, Bretthauer M. Effect of flexible sigmoidoscopy screening on colorectal cancer incidence and mortality: a randomized clinical trial. *JAMA*. Aug 13 2014;312(6):606-15. doi:10.1001/jama.2014.8266

47. Yang K, Cao Y, Liu Y, Gurjao C. CLINICAL AND GENOMIC CHARACTERIZATION OF INTERVAL COLORECTAL CANCER IN THREE PROSPECTIVE COHORTS. Conference Abstract. *Gastroenterology*. 2022;162(7):S-188-S-189. doi:10.1016/S0016-5085(22)60451-7

48. Zgraggen A, Stoffel ST, Barbier MC, Marbet UA. Colorectal cancer surveillance by colonoscopy in a prospective, population-based long-term Swiss screening study - outcomes, adherence, and costs. *Z Gastroenterol*. May 2022;60(5):761-778. Langzeituberwachung nach dem kolorektalen Karzinomscreening mittels Koloskopie in einer prospektiven Bevolkerungsstudie in der Schweiz: Resultate, Adharenz und Kosten. doi:10.1055/a-1796-2471

49. Wang K, Ma W, Wu K, et al. Healthy lifestyle, endoscopic screening, and colorectal cancer incidence and mortality in the United States: A nationwide cohort study. *PLoS Med*. Feb 2021;18(2):e1003522. doi:10.1371/journal.pmed.1003522

50. Laiyemo A, Kibreab A, Scott V, et al. Colorectal cancer screening uptake among deaf adults in the United States. Conference Abstract. *American Journal of Gastroenterology*. 2021;116(SUPPL):S144. doi:10.14309/01.ajg.0000773796.13425.15

51. Abdel-Rahman O. Patterns and Trends of Cancer Screening in Canada: Results From a Contemporary National Survey. *J Natl Compr Canc Netw*. Jan 6 2021;19(1):68-76. doi:10.6004/jnccn.2020.7613

52. Okereke I, Anderson S, cherfrere C, Okon E, Scott VF. THE ASSOCIATION OF PATIENTSâ€™ PERCEPTION OF THEIR HEALTH STATUS AND COLORECTAL CANCER SCREENING UPTAKE AMONG US ADULTS. Conference Abstract. *Gastroenterology*. 2020;158(6):S-915. doi:10.1016/S0016-5085(20)32974-7

53. Ezeofor A, McDonald-Pinkett S, Laiyemo AO. THE ASSOCIATION OF PATIENTSâ€™ PERCEPTION OF HEALTHCARE PROVIDERSâ€™ COMMUNICATIONS QUALITY AND COLORECTAL CANCER SCREENING UPTAKE AMONG US ADULTS. Conference Abstract. *Gastroenterology*. 2020;158(6):S-1178. doi:10.1016/S0016-5085(20)33614-3

54. Peterse EF, Meester R, Siegel R, Chen J, Dwyer A, Ahnen D. Colorectal Cancer Screening Initiation at Age 45 Years: A Microsimulation Analysis to Address the Rising Incidence in Young Adults. Conference Abstract. *Gastroenterology*. 2018;154(6):S-71-S-72. doi:10.1016/S0016-5085(18)30692-9

55. Peterse EFP, Meester RGS, Siegel RL, et al. The impact of the rising colorectal cancer incidence in young adults on the optimal age to start screening in the US: A microsimulation analysis. Conference Abstract. *Journal of Global Oncology*. 2018;4:46s. doi:10.1200/jgo.18.34900

56. Nozaki R, Yamada K, Takano M. Reduction in the risk of mortality by performing mass screening for colorectal cancer using fecal occult blood testing in combination with flexible sigmoidoscopy. Conference Abstract. *Journal of gastroenterology and hepatology*. 2017;32:108. doi:10.1111/jgh.13877

57. Click BH, Doroudi M, Hickey T, Pinsky P, Schoen RE. Long term risk of colorectal cancer after detection of adenomatous polyps. Conference Abstract. *Gastroenterology*. 2017;152(5):S179.

58. Lieberman D, Abbott DH, O'Leary MC, Hauser ER, Williams C. Clinical risk group at baseline is associated with 10 year outcomes in a screening cohort-longitudinal analysis of the CSP 380 cohort. Conference Abstract. *Gastroenterology*. 2016;150(4):S184.

59. Tang RS, Wong MC, Ching J, Shum JP, Lam TY, Ng SC. Comparison of the performance of flexible sigmoidoscopy alone and combined flexible sigmoidoscopy and fecal immunochemical test as colorectal cancer screening tool in 5406 asymptomatic Chinese adults. Conference Abstract. *Gastroenterology*. 2015;148(4):S160-S161.

60. Keller SC, Momplaisir F, Lo Re V, et al. Colorectal cancer incidence and screening in US Medicaid patients with and without HIV infection. *AIDS Care*. 2014;26(6):716-22. doi:10.1080/09540121.2013.855700

61. Razzak A, Yu K, Pinsky P, Riley T, Schoen RE. Association between family history of colorectal cancer and incident colorectal cancer in the PLCO trial. Conference Abstract. *Gastroenterology*. 2014;146(5):S162-S163. doi:10.1016/S0016-5085(14)60581-3

62. Mehta SJ, Jensen CD, Ghai NR, et al. The effect of an organized screening program on racial and ethnic disparities and choice of colorectal cancer screening test. Conference Abstract. *Gastroenterology*. 2014;146(5):S168-S169. doi:10.1016/S0016-5085(14)60598-9

63. Doubeni CA, Weinmann S, Adams KF, et al. Screening colonoscopy and risk of incident late-stage colorectal cancer diagnosis in average-risk adults: A nested case-control study. Conference Abstract. *Gastrointestinal endoscopy*. 2013;77(5):AB430.

64. Wu BU, Ngor EW. Effectiveness of flexible sigmoidoscopy versus colonoscopy for prevention of colorectal cancer: Implications of a negative initial screening examination. Conference Abstract. *Gastroenterology*. 2013;144(5):S204-S205.

65. Jeffers K, Burnside C, Sanderson A, Polston E, Begum R, Nunlee-Bland G. An analysis of colorectal cancer screening behavior among overweight and obese participants in a national survey. Conference Abstract. *American Journal of Gastroenterology*. 2012;107:S796. doi:10.1038/ajg.2012.279

66. Courtney RJ, Paul CL, Sanson-Fisher RW, et al. Colorectal cancer screening in Australia: a community-level perspective. *Med J Aust*. May 7 2012;196(8):516-20. doi:10.5694/mja11.10661

67. Nishihara R, Lochhead P, Wu K, et al. Long-term risk of colorectal cancer risk after lower endoscopy and polypectomy. Conference Abstract. *Gastroenterology*. 2012;142(5):S111.

68. Doria-Rose VP, Newcomb PA, Levin TR, Conell C, Weiss N. Risk factors for distal colon and rectal cancer following a negative screening sigmoidoscopy. Conference Abstract. *Gastroenterology*. 2011;140(5):S406. doi:10.1016/S0016-5085(11)61666-1

69. Burnet-Hartman A, Newcomb P, Phipps A, et al. Sigmoidoscopy and colonoscopy are inversely associated with both left- and right-sided advanced adenomas. Conference Abstract. *Cancer Epidemiology Biomarkers and Prevention*. 2011;20(4):716. doi:10.1158/1055-9965.EPI-11-0086

70. Aggarwal A, Lee A, Kazis L, Dan B. USE of colorectal cancer screening modalities in patients with chronic mental llnesses. Conference Abstract. *Journal of Investigative Medicine*. 2011;59(2):535. doi:10.231/JIM.0b013e31820bab4c

71. Anand N, Ottaway C, Irvine E. A case-control study to assess the risk of colonic neoplasms in diabetic and non-diabetic patients undergoing colonoscopy. Conference Abstract. *Canadian Journal of Gastroenterology*. 2009;23

72. Farraye FA, Wong M, Hurwitz S, et al. Barriers to endoscopic colorectal cancer screening: are women different from men? *The American journal of gastroenterology*. Feb 2004;99(2):341-9. doi:10.1111/j.1572-0241.2004.04045.x

73. Holme O, Loberg M, Kalager M, Bretthauer M, Aas E, Hoff G. Effect of flexible sigmoidoscopy on incidence and mortality from colorectal cancer; first largescale populaton-based trial. Conference Abstract. *United European Gastroenterology Journal*. 2013;1(1):A69. doi:10.1177/2050640613502899

74. Atkin WS, Cuzick J, Duffy SW, et al. UK flexible sigmoidoscopy screening trial: Colorectal cancer incidence and mortality rates at 11 years after a single screening examination. Conference Abstract. *Gastroenterology*. 2010;138(5):S53.

75. Newcomb PA, Norfleet RG, Storer BE, Surawicz TS, Marcus PM. Screening sigmoidoscopy and colorectal cancer mortality. Article. *Journal of the National Cancer Institute*. 1992;84(20):1572-1575.
