# Supplementary material for: Is flexible sigmoidoscopy screening associated with reducing colorectal cancer incidence and mortality? a meta-analysis and systematic review
Source: Front Oncol. 2023 Dec 13;13:1288086. doi: 10.3389/fonc.2023.1288086 (PMC10757863; doi:10.3389/fonc.2023.1288086)
Supplement: Supplementary file 5 [file Table_5.docx]

**Supplementary Table 5. Study Quality Assessment for Cohort Studies (Newcastle-Ottawa Scale)**

| Study | Selection | | | |  | Outcome | | |  |
| --- | --- | --- | --- | --- | --- | --- | --- | --- | --- |
|  | Expoesd Cohort | Noexposed Cohort | Ascertainment of Exposure | Outcome of Interest | Comparability | Assessment of Outcome | Length of Follow-Up | Adequacy of Follow-Up | Total Number of Stars |
| Wu et al., 2014 | * | * | - | * | ** | * | * | * | 8 |
