# Supplementary material for: Is flexible sigmoidoscopy screening associated with reducing colorectal cancer incidence and mortality? a meta-analysis and systematic review
Source: Front Oncol. 2023 Dec 13;13:1288086. doi: 10.3389/fonc.2023.1288086 (PMC10757863; doi:10.3389/fonc.2023.1288086)
Supplement: Supplementary file 6 [file Table_6.docx]

**Supplementary Table 6. Study Quality Assessment for *Jadad* score (original version)**

| Study | Was the study described as randomized | | Was the study described as double blind | | Was there a description of withdrawals and dropouts | Total score |
| --- | --- | --- | --- | --- | --- | --- |
|  | Method of randomization was described and it was appropriate | Method of randomization was inappropriate | Method of blinding was described and it was appropriate | Method of blinding was inappropriate |  |  |
| Senore et al., 2022 | 2 | 0 | 0 | 0 | 1 | 3 |
| Miller et al., 2019 | 2 | 0 | 0 | 0 | 1 | 3 |
| Holme et al., 2018 | 2 | 0 | 0 | 0 | 1 | 3 |
| Atkin et al., 2017 | 2 | 0 | 0 | 0 | 1 | 3 |
| Thiis-Evensen et al., 2013 | 2 | -1 | 0 | 0 | 1 | 2 |
| Randel et al., 2020 | 2 | 0 | 0 | 0 | 1 | 3 |
