# Supplementary material for: Is flexible sigmoidoscopy screening associated with reducing colorectal cancer incidence and mortality? a meta-analysis and systematic review
Source: Front Oncol. 2023 Dec 13;13:1288086. doi: 10.3389/fonc.2023.1288086 (PMC10757863; doi:10.3389/fonc.2023.1288086)
Supplement: Supplementary file 7 [file Table_7.docx]

**Supplementary Table 7. Quality of Evidence of Primary Outcomes**

| **FS compared to Non FS for adults over 18 years old without CRC** | | | | | | |
| --- | --- | --- | --- | --- | --- | --- |
| **Patient or population:** patients with adults over 18 years old without CRC  **Settings:** Randomized trials and observational study  **Intervention:** FS  **Comparison:** Non FS | | | | | | |
| **Outcomes** | **Illustrative comparative risks* (95% CI)** | | **Relative effect**  **(95% CI)** | **No of Participants**  **(studies)** | **Quality of the evidence**  **(GRADE)** | **Comments** |
|  | Assumed risk | Corresponding risk |  |  |  |  |
|  | **Non FS** | **FS** |  |  |  |  |
| **Incidence in RCT study**  Follow-up: median 16.55 years | **Study population** | | **RR 0.74**  (0.66 to 0.84) | 563978  (6 studies) | ⊕⊕⊕⊕  **high** |  |
|  | **21 per 1,000** | **16 per 1,000**  (14 to 18) |  |  |  |  |
|  | **Moderate** | |  |  |  |  |
|  | **25 per 1,000** | **19 per 1,000**  (17 to 21) |  |  |  |  |
| **Incidence in Cohort study**  Follow-up: median 11 years | **Study population** | | **RR 3.57**  (2.39 to 5.32) | 138297  (1 study) | ⊕⊕⊕⊝  **moderate**^1^ |  |
|  | **1 per 1,000** | **2 per 1,000**  (2 to 3) |  |  |  |  |
|  | **Moderate** | |  |  |  |  |
|  | **1 per 1,000** | **4 per 1,000**  (2 to 5) |  |  |  |  |
| **Mortality in RCT study**  Follow-up: median 18.26 years | **Study population** | | **RR 0.7**  (0.58 to 0.85) | 424687  (5 studies) | ⊕⊕⊕⊕  **high** |  |
|  | **8 per 1,000** | **5 per 1,000**  (5 to 7) |  |  |  |  |
|  | **Moderate** | |  |  |  |  |
|  | **9 per 1000** | **6 per 1000**  (5 to 8) |  |  |  |  |
| *The basis for the **assumed risk** (e.g. the median control group risk across studies) is provided in footnotes. The **corresponding risk** (and its 95% confidence interval) is based on the assumed risk in the comparison group and the **relative effect** of the intervention (and its 95% CI).  **CI:** Confidence interval; **RR:** Risk ratio; | | | | | | |
| GRADE Working Group grades of evidence  **High quality:** Further research is very unlikely to change our confidence in the estimate of effect.  **Moderate quality:** Further research is likely to have an important impact on our confidence in the estimate of effect and may change the estimate.  **Low quality:** Further research is very likely to have an important impact on our confidence in the estimate of effect and is likely to change the estimate.  **Very low quality:** We are very uncertain about the estimate. | | | | | | |
| ^1^ Effect size RR > 2 | | | | | | |
